# High out-of-plane piezoelectric response in Janus SeWZH (Z = N, P, As) semiconductors: a first-principles prediction

**DOI:** 10.1039/d6ra05801a

**Published:** 2026-09-10

**Authors:** A. I. Kartamyshev, Vo Q. Nha, Le D. Hieu, Tuan V. Vu, Pham T. Truong, Huynh V. Phuc, Nguyen T. Hiep

**Affiliations:** a Laboratory for Computational Physics, Institute for Computational Science and Artificial Intelligence, Van Lang University Ho Chi Minh City Vietnam andrey.kartamyshev@vlu.edu.vn; b Faculty of Mechanical, Electrical, and Computer Engineering, Van Lang School of Technology, Van Lang University Ho Chi Minh City Vietnam; c School of Engineering and Technology, Hue University Hue Vietnam voquangnha@hueuni.edu.vn; d Division of Physics, School of Education, Dong Thap University Dong Thap 870000 Vietnam; e Institute of Research and Development, Duy Tan University Da Nang 550000 Vietnam nguyenthihiep2@duytan.edu.vn; f Faculty of Natural Science, Duy Tan University Da Nang 550000 Vietnam

## Abstract

A systematic first-principles investigation has been conducted to explore the fundamental properties of Janus SeWZH (Z = N, P, As) monolayers. Cohesive-energy calculations, phonon-dispersion analysis, and *ab initio* molecular dynamics simulations consistently verify the energetic, dynamical, and thermal stabilities of the proposed SeWNH, SeWPH, and SeWAsH monolayers, indicating their potential feasibility for experimental synthesis. Electronic structure calculations reveal semiconducting behavior for all SeWZH monolayers, with band gaps between 1.59 and 2.61 eV at the HSE06 theoretical level. Specifically, SeWNH is identified as an indirect-band-gap semiconductor, whereas SeWPH and SeWAsH possess direct band gaps. Structural asymmetry induces in-plane and out-of-plane piezoelectricity, with the highest out-of-plane coefficient *d*_31_ = 0.51 pm V^−1^ obtained for SeWNH. Furthermore, spin–orbit coupling drives significant spin splitting at the valence band *K* valley. Charge transport analysis reveals that acoustic deformation potential scattering dominates, resulting in low carrier mobilities. The predicted low carrier mobilities reflect the strong influence of intrinsic scattering on charge transport.

## Abstract 2

A systematic first-principles investigation has been conducted to explore the fundamental properties of Janus SeWZH (Z = N, P, As) monolayers.

## Graphical abstract

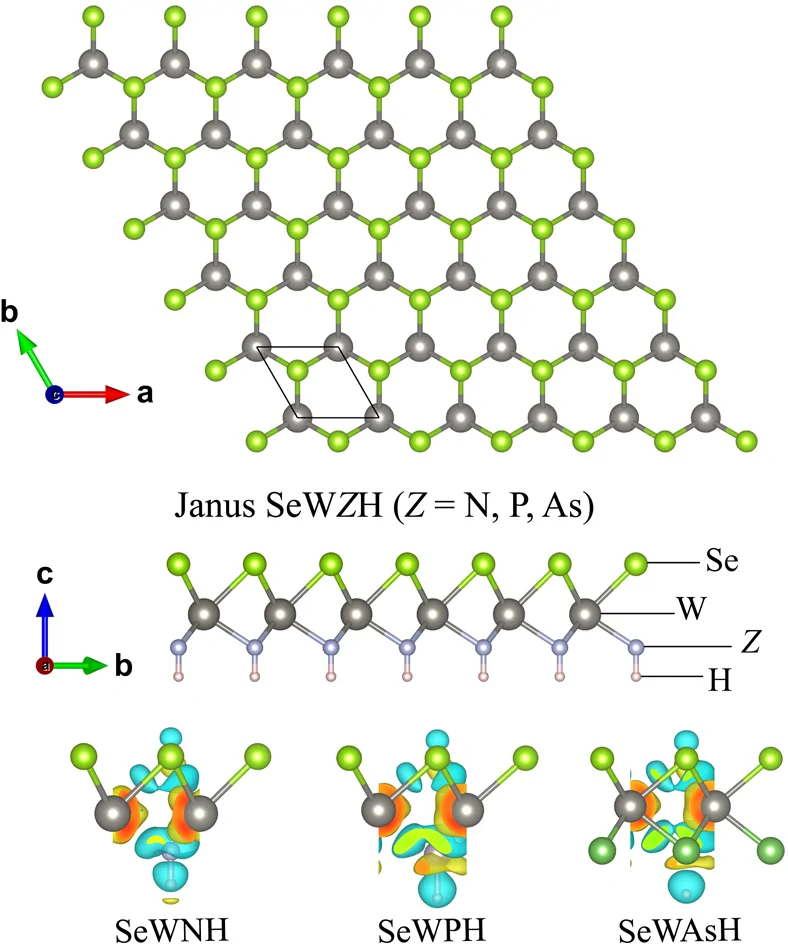

## 1 Introduction

The emergence of Janus two-dimensional (2D) materials has opened new opportunities for designing multifunctional nanomaterials with broken structural symmetry. Unlike conventional 2D crystals, Janus structures are formed by replacing atoms on one surface with different elements, thereby eliminating the mirror symmetry perpendicular to the layer.^1–3^ Such an asymmetric configuration induces a built-in electric field and spontaneous polarization, leading to distinctive piezoelectric behavior. As a result, these materials exhibit piezoelectric responses not only within the atomic plane but also along the out-of-plane direction, which is generally inaccessible in symmetric monolayers.^4–6^ These characteristics make Janus materials attractive for a broad range of applications, including wearable electronics, microelectromechanical systems, sensors, and energy-conversion devices. Since the successful prediction of Janus MoSSe, extensive theoretical and experimental efforts have revealed enhanced piezoelectric coefficients in numerous Janus systems, including MoSSe, WSSe, MoSTe, and WSTe monolayers.^7–10^ More recently, novel Janus compounds containing heavy elements have attracted considerable interest because the combination of strong spin–orbit coupling and large piezoelectric polarization may enable multifunctional devices that integrate electromechanical and spintronic functionalities.^11,12^ These developments continue to motivate the exploration of novel Janus materials with improved piezoelectric performance.

In addition to piezoelectricity, charge-transport properties are another key factor in determining the performance of 2D materials in electronic and optoelectronic applications. The intrinsic structural asymmetry of Janus structure also modifies electron–phonon interactions, leading to transport characteristics that differ substantially from those of conventional symmetric monolayers.^13–15^ Accurate prediction of carrier mobility in these materials requires a detailed understanding of the scattering processes that govern charge transport. Although deformation-potential theory has been widely adopted for estimating carrier mobility in semiconductors, it often neglects several important scattering mechanisms and may therefore overestimate transport performance.^16–18^ To overcome these limitations, the recently developed AMSET (*Ab initio* Model for Mobility and Seebeck Coefficient using the Boltzmann Transport Equation) framework provides a more rigorous first-principles approach by explicitly incorporating multiple scattering channels, including acoustic deformation potential (ADP), polar optical phonon (POP), ionized impurity (IMP), and piezoelectric (PIE) scattering.^19–21^ This methodology enables the identification of the dominant mobility-limiting mechanism and offers a more realistic description of carrier dynamics in semiconductors. AMSET-based studies have demonstrated that different scattering processes can dominate carrier transport depending on the crystal structure and carrier concentration of the material.^6,22,23^ Therefore, the AMSET analysis not only provides reliable carrier-mobility predictions but also reveals the microscopic mechanisms governing charge transport, thereby facilitating the design of high-performance Janus materials for nanoelectronic, optoelectronic, and spintronic applications.

From the above motivations, a systematic study of piezoelectric behavior, electronic band structures, and AMSET-predicted carrier mobilities is crucial for establishing the structure and property relationships in Janus 2D materials and assessing their potential for advanced electromechanical, spintronic, electronic and energy-conversion applications. Here, based on density functional theory (DFT), we introduce Janus SeWZH (Z = N, P, As) monolayers as new asymmetric 2D materials and investigate their physical properties. To establish the viability of proposed structures, we evaluate their energetic, dynamical, and thermal stabilities through cohesive-energy analysis, phonon-dispersion calculations, and *ab initio* molecular dynamics (AIMD) simulations. Mechanical properties with elastic constants, Young's modulus and Poisson's ratio are also examined to assess their structural robustness. The piezoelectric response of the SeWZH monolayers is also explored by determining their piezoelectric coefficients along both the in-plane and out-of-plane directions. To reveal the electronic properties of these materials, we perform a comprehensive study of electronic band structures employing both the Heyd–Scuseria–Ernzerhof (HSE06) as well as Perdew–Burke–Ernzerhof (PBE) methods, including the effects of spin–orbit coupling. Moreover, charge transport properties are studied by using the AMSET framework to evaluate the contributions of different scattering channels to the overall electron and hole mobilities.

## 2 Computational methods

Density functional theory simulations were performed using VASP.^24,25^ Core–valence interactions were described by the PAW formalism,^26,27^ with a plane-wave cutoff energy of 650 eV. Brillouin-zone integrations employed a 12 × 12 × 1 Monkhorst–Pack sampling scheme, while a vacuum spacing of 25 Å along the surface-normal direction ensured negligible interlayer interactions. All structures were fully relaxed until the Hellmann–Feynman forces on each atom were below 10^−3^ eV Å^−1^, and the electronic energy converged to 10^−6^ eV. To eliminate artifacts associated with the intrinsic polarity of the Janus monolayers, dipole corrections were applied along the out-of-plane direction. Phonon dispersions were calculated using the finite-displacement approach as implemented in PHONOPY with a 5 × 5 × 1 supercell. Thermal stability was validated by AIMD simulations in the canonical ensemble at 300 K. Exchange–correlation effects were treated using the PBE functional,^28^ whereas the HSE06 hybrid functional^29^ was adopted for accurate electronic structure predictions. Spin–orbit coupling was incorporated self-consistently owing to its significant influence on the band topology and electronic states of heavy-element-containing systems.^30^ Elastic and piezoelectric responses were obtained within the density functional perturbation theory framework. Charge-transport properties were subsequently investigated using the AMSET package,^19^ which evaluates carrier mobility through a comprehensive treatment of polar optical phonon, acoustic deformation potential, ionized impurity, and piezoelectric scattering mechanisms.

## 3 Results and discussion

### 3.1 Lattice parameters and stabilities

**Fig. 1:**
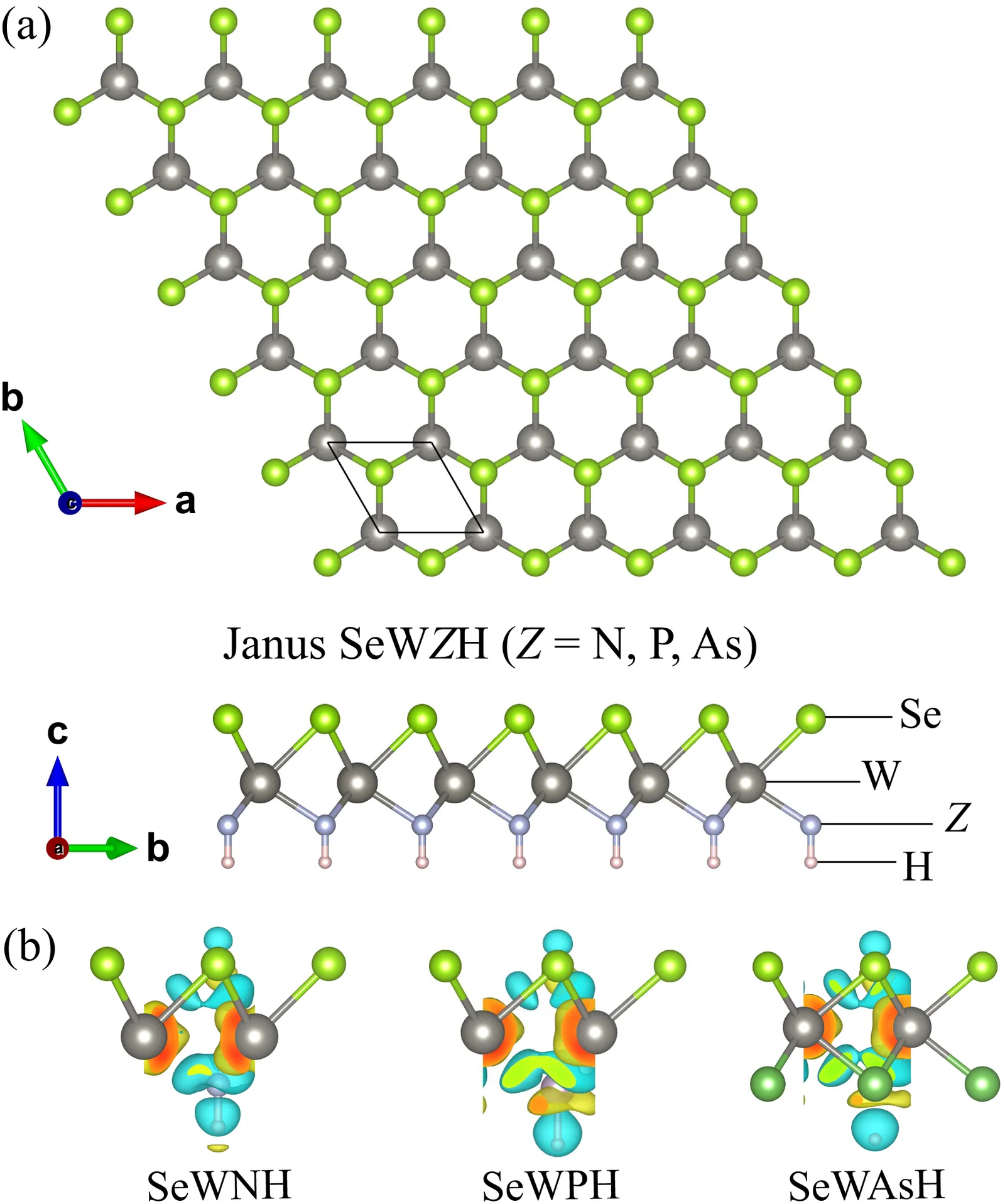
(a) Top/side views and (b) the charge density difference of SeWZH (Z = N, P, As) structures.

The crystal structure and stability of the SeWZH (Z = N, P, As) materials are first analyzed to evaluate their feasibility for experimental realization. As illustrated in Fig. 1(a), the optimized crystal structure of the proposed SeWZH monolayers crystallize in a hexagonal lattice geometry within the *P*3*m*1 space group. The monolayers are constructed from four stacked sublayers with asymmetric arrangement, where the Z–W is located between the outer H and Se atomic sheets. The corresponding optimized structural parameters summarized in Table 1 reveal a systematic enlargement of the lattice constant *a* from 3.09 Å to 3.38 Å as the Z changes from N to As. A similar increasing tendency is observed for both the bond lengths and monolayer thickness. This trend originates from the progressive increase in the atomic radius of the pnictogen element from N to P and As. As a result, the equilibrium bond distances become larger, leading to a gradual expansion of the SeWZH lattice.

**Table 1:** Lattice parameter *a* (Å), chemical bond lengths *d* (Å), layer thickness Δ*h* (Å), atom Bader charge *q* (in unit of *e*), cohesive energy *E*_coh_ (eV per atom), and formation energy *E*_form_ of Janus SeWZH structures

	*a*	*d*_Se–W_	*d*_W–Z_	*d*_Z–H_	Δ*h*	*q*_Se_	*q*_W_	*q*_Z_	*q*_H_	*E*_coh_	*E*_form_
SeWNH	3.09	2.51	2.13	1.02	3.94	0.850	0.194	−0.061	−0.984	−7.10	−7.07
SeWPH	3.30	2.55	2.39	1.42	4.55	−0.288	0.265	0.578	−0.554	−6.35	−2.57
SeWAsH	3.38	2.56	2.49	1.52	4.72	−0.324	0.195	0.404	−0.275	−5.96	−1.72

Next, the charge density difference maps are calculated to visualize how electrons are redistributed in the SeWZH monolayers as shown in Fig. 1(b). Consistent with the Bader charge analysis as listed in Table 1, the charge density difference visualization reveals that charge is depleted around the W atoms (indicated by the positive Bader charge) and accumulates around the H atoms (indicated by their negative Bader charges). The calculated Bader charges of the H atoms decrease from −0.984*e*, −0.554*e*, to −0.275*e* for SeWNH, SeWPH, and SeWAsH, respectively. The gradual reduction in charge transfer from N to As suggests that the interaction between the H layer and the rest of the monolayer becomes weaker as the size of the pnictogen atom Z increases.

The dynamical stability of the SeWZH monolayers is evaluated through their cohesive energies (*E*_coh_). This parameter quantifies the energy gain associated with crystal formation from isolated atoms and serves as a fundamental measure of bond strength and structural robustness. It is defined as:1
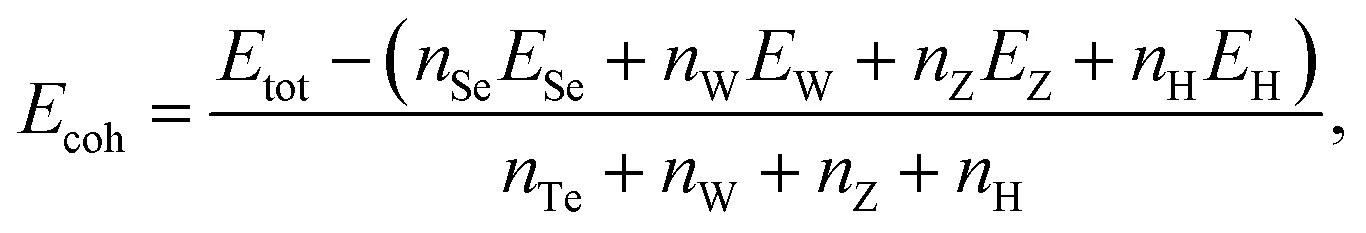
where *E*_tot_ denotes the total energy of the SeWZH monolayer, while *E*_Se_, *E*_W_, *E*_Z_, *E*_H_ and *n*_Se_, *n*_W_, *n*_Z_, *n*_H_ represent the energy and number of the corresponding isolated atomic species, respectively. More negative values of *E*_coh_ indicate stronger atomic interactions and enhanced dynamical stability.

Table 1 shows that the calculated cohesive energies of SeWNH, SeWPH, and SeWAsH are −7.10, −6.35, and −5.96 eV per atom, respectively. The less negative *E*_coh_ values from SeWNH to SeWAsH is observed, indicating that the atomic bonding becomes weaker as the pnictogen atom changes from N to As. Among the three monolayers, SeWNH has the lowest cohesive energy, implying the strongest atomic bonding and the greatest energetic stability. This observation is consistent with its compact crystal structure and the shortest bond lengths.

Furthermore, the formation energies of the proposed materials were carried out to assess their thermodynamic stability and tendency toward phase separation. This quantity measures the energetic favorability of the proposed structures relative to their constituent elements in their standard reference states, thereby providing an important criterion for evaluating their potential experimental realizability under practical synthesis conditions. The formation energy *E*_form_ of SeWZH is expressed as:^31,32^

2 *E*_form_ = *E*_tot_ − (*µ*_Se_ + *µ*_W_ + *µ*_Z_ + *µ*_H_),

where *E*_tot_ is the total energy of the Janus SeWZH, and *µ*_Se_, *µ*_W_, *µ*_Z_, and *µ*_H_ denote the chemical potentials of Se, W, Z, and H, respectively.

As summarized in Table 1, the calculated formation energies for the SeWNH, SeWPH, and SeWAsH monolayers are −7.07, −2.57, and −1.72 eV, respectively. These negative values confirm that the materials possess intrinsic thermodynamic stability relative to their constituent elements. Such robust energetic viability suggests that the proposed structures are highly promising for experimental realization, potentially through precise techniques.

**Fig. 2:**
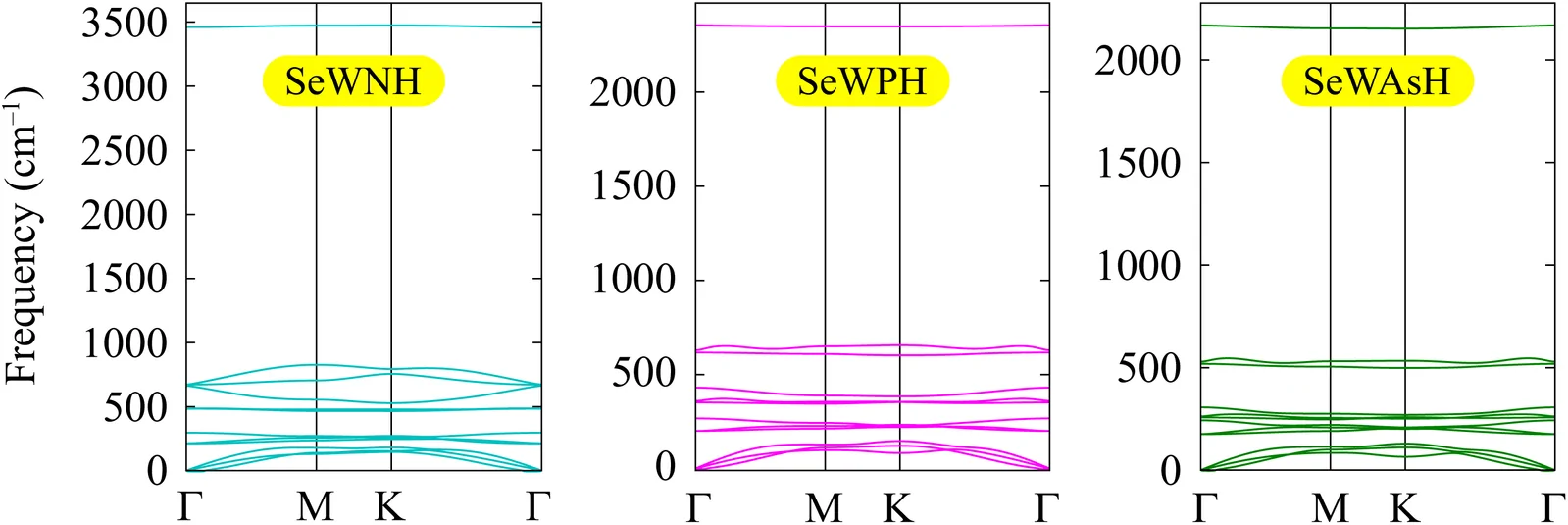
Calculated phonon dispersion relations for the two-dimensional Janus SeWZH (Z = N, P, As) monolayers.

To definitively establish the dynamic stability of the proposed structures, the phonon dispersion spectra for the SeWNH, SeWPH, and SeWAsH monolayers were calculated and are depicted in Fig. 2. The spectra demonstrate the robust dynamic stability of these proposed materials. At the center of the Brillouin zone, the expected three acoustic branches originate from zero frequency at the *Γ* point. It is simply worth noting that a negligibly small negative frequency appears strictly in the immediate vicinity of the *Γ* point, corresponding to the out-of-plane flexural acoustic (ZA) mode. As established in the computational study of 2D materials,^33^ such minor imaginary frequencies are common numerical artifacts resulting from finite supercell sizes and computational limits in perfect translational invariance, rather than an indication of structural instability.

**Fig. 3:**
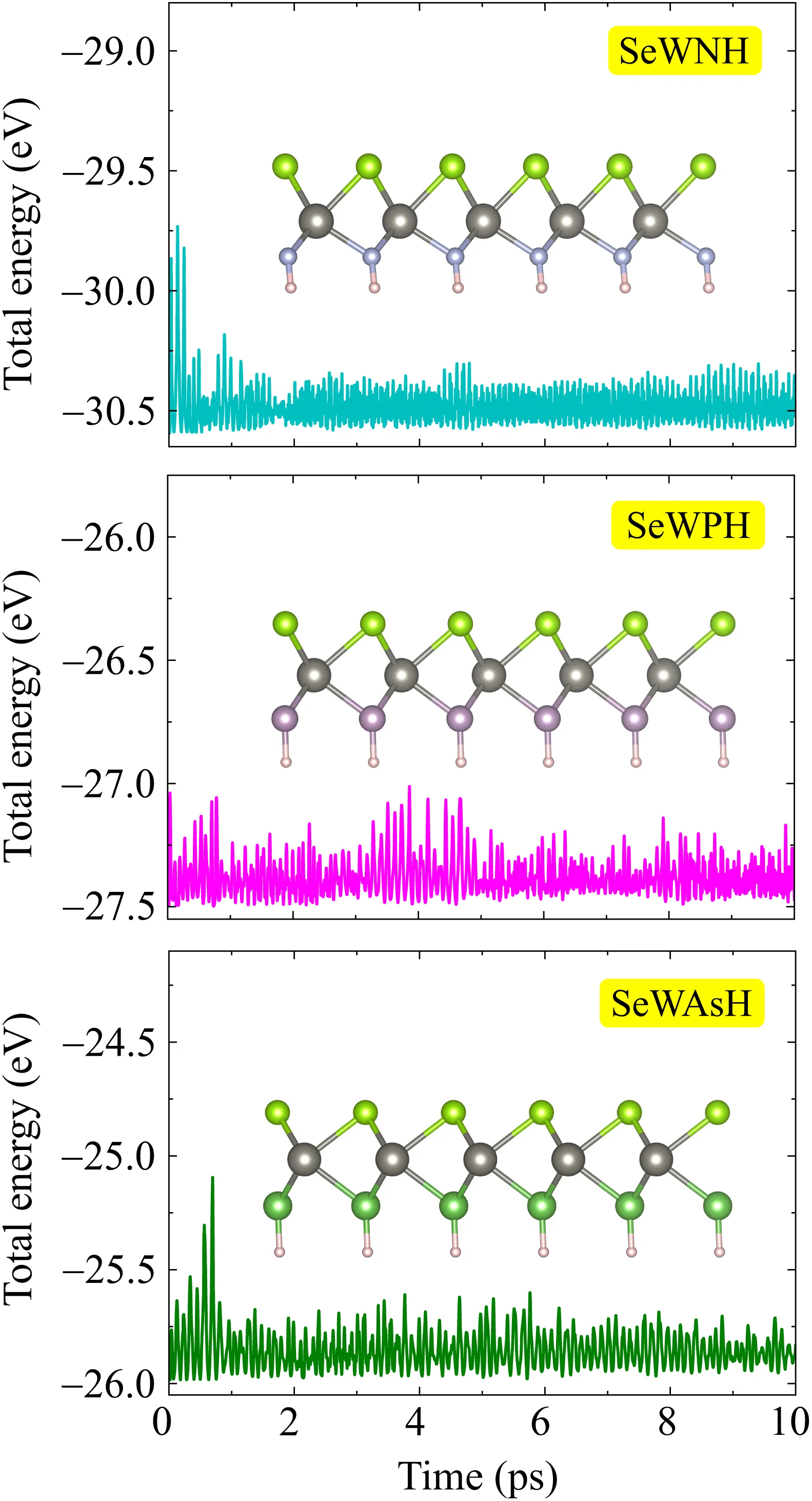
Fluctuations in the total energy with heating time from AIMD calculations of SeWZH monolayers. Insets are the corresponding SeWNH, SeWPH, SeWAsH structures after the simulation.

Following the verification of dynamical stability, we further assess the thermal stability of the SeWZH monolayers using AIMD simulations. The calculations are performed at 300 K for 10 ps with a simulation time step of 1 fs. Fig. 3 displays the corresponding total energy profiles, which show only slight fluctuations during the entire simulation period, suggesting that thermal perturbations have a limited impact on the SeWZH systems. Examination of the final AIMD snapshots reveals that the crystal lattices remain essentially unchanged, with no observable bond breaking or significant structural distortion. These findings provide strong evidence that the proposed Janus SeWZH monolayers possess excellent thermal stability and can remain structurally intact under ambient conditions.

### 3.2 Mechanical and piezoelectric properties

The mechanical stability and piezoelectric properties of the SeWZH monolayers are subsequently examined to evaluate their potential for practical device integration. Mechanical parameters such as the elastic constants, the in-plane Young's modulus (*Y*_2D_) and Poisson's ratio (*ν*_2D_) are useful for understanding the resistance of the material to external deformation and its overall structural flexibility. Owing to the hexagonal symmetry of the SeWZH structures, only two independent elastic constants of *C*_11_ and *C*_12_, need to be determined, while *C*_22_ = *C*_11_ and *C*_66_ = (*C*_11_ − *C*_12_)/2. These elastic stiffness coefficients are obtained by introducing small strains from −1.5 to 1.5% along the in-plane directions and fitting the calculated total-energy variations as a function of strain.^5^ The calculated elastic parameters are summarized in Table 2. The values of *C*_11_ range from 101.34 to 173.15 N m^−1^, whereas *C*_12_ and *C*_66_ are much smaller varying from 20.58 to 33.67 N m^−1^ and 40.33 to 69.63 N m^−1^, respectively. Importantly, all elastic constants are positive and satisfy the Born–Huang stability requirement, *C*_11_ − *C*_12_ > 0, for two-dimensional hexagonal crystals.^34,35^ These findings confirm the excellent mechanical stability of SeWNH, SeWPH, SeWAsH monolayers and suggest their feasibility for experimental synthesis.

**Table 2:** Elastic coefficient *C*_*ij*_, Young's modulus *Y*_2D_, Poisson's ratio *ν*_2D_, and piezoelectric coefficients (*e*_11_, *e*_31_, *d*_11_, *d*_31_) for Janus SeWZH monolayers

	*C*_11_, (N m^−1^)	*C*_12_, (N m^−1^)	*C*_66_, (N m^−1^)	*Y*_2D_, (N m^−1^)	*ν*	*e*_11_, 10^−10^ C m^−1^	*e*_31_, 10^−10^ C m^−1^	*d*_11_, (pm V^−1^)	*d*_31_, (pm V^−1^)
SeWNH	173.15	33.67	69.63	166.74	0.19	2.76	1.04	1.98	0.51
SeWPH	111.82	20.76	45.45	108.07	0.19	3.90	0.13	4.29	0.1
SeWAsH	101.34	20.58	40.33	97.26	0.20	4.38	−0.07	5.42	−0.06

The obtained elastic constants above could be used to determine additional mechanical parameters that describe the response of SeWZH monolayers to external stress. In particular, the two-dimensional Young's modulus (*Y*_2D_) describes the stiffness of monolayers against in-plane stretching, whereas the Poisson's ratio (*ν*_2D_) reflects their transverse deformation accompanying an applied tensile. These parameters are calculated as follows:3
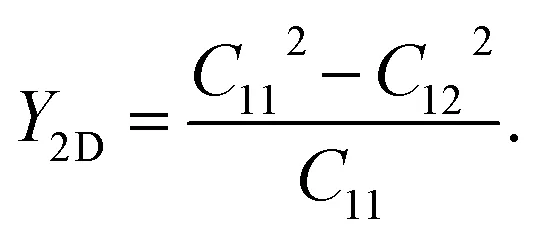
4
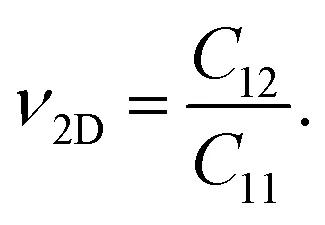


As listed in Table 2, the calculated Young's modulus decreases from 166.74 N m^−1^ for SeWNH to 97.26 N m^−1^ for SeWAsH, indicating a reduction in lattice stiffness with increasing pnictogen atomic size. Compared with well-known 2D materials such as graphene (*Y*_2D_ = 336 N m^−1^),^36^ MoSi_2_P_4_ (*Y*_2D_ = 203.00 N m^−1^),^37^ and ZrGeN_3_H (*Y*_2D_ = 217.88 N m^−1^),^38^ the investigated SeWZH monolayers exhibit significantly lower stiffness and therefore greater mechanical flexibility. Meanwhile, the calculated Poisson's ratio of the three monolayers remains relatively low of 0.19 to 0.20, as summarized in Table 1. These low *ν*_2D_ values imply limited transverse contraction under tensile loading, reflecting the strong chemical bonding within the SeWZH lattice.

Furthermore, the piezoelectric characteristics of the SeWZH monolayers are also explored to determine their potential in nanoscale piezoelectric devices. When a piezoelectric crystal is subjected to mechanical strain, the redistribution of charges within the lattice generates an electric polarization. This electromechanical coupling enables the direct conversion between mechanical and electrical energy. Following the density functional theory methodology reported by Duerloo and co-workers,^5^ we calculate the piezoelectric response of the investigated SeWZH monolayers. The piezoelectric coefficients *e*_*ijk*_ and *d*_*ijk*_ are evaluated from the polarization changes induced by external strain according to the equations below:^39^5
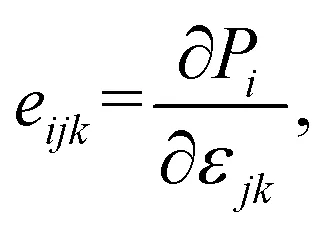
6
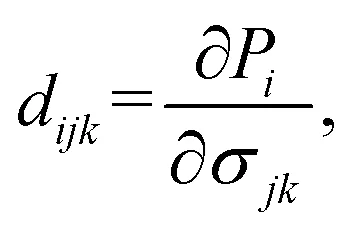
in which *σ*_*jk*_ and *ε*_*jk*_ refer to the stress and strain tensor components, respectively, with the subscripts (*i*, *j*, *k*) labelling the Cartesian directions (*x*, *y*, *z*). Owing to the atomically thin layer of the investigated Janus SeWZH materials, the piezoelectric coefficients obtained from three-dimensional calculations are rescaled to remove the artificial contribution from the vacuum region. The corresponding two-dimensional piezoelectric coefficients are therefore determined according to *e*^2D^_*ij*_ = *z*, *e*^3D^_*ij*_, where *z* represents the out-of-plane cell dimension.

Based on the calculated values of the piezoelectric stress tensor *e*_*ij*_, the corresponding piezoelectric strain coefficients *d*_*ij*_ can be evaluated using the following expression, which relates the electrical and mechanical responses of the material:^39^7
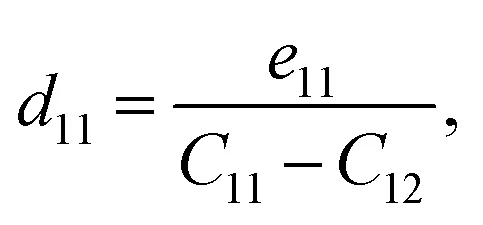
8
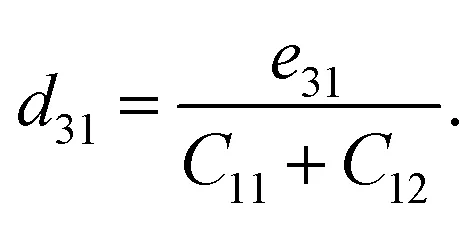


A notable feature of the SeWZH monolayers is their excellent piezoelectric performance, as evidenced by the calculated coefficients summarized in Table 3. The in-plane piezoelectric strain coefficients *d*_11_ are calculated to be 1.98, 4.29, and 5.42 pm V^−1^ for SeWNH, SeWPH, and SeWAsH, respectively. These values are substantially larger than those of many conventional two-dimensional piezoelectric materials such as h-BN (*d*_11_ = 0.60 pm V^−1^),^5^ GaP (*d*_11_ = 0.96 pm V^−1^),^40^ GaO (*d*_11_ = 0.41 pm V^−1^).^41^ This highlights the strong electromechanical coupling in this SeWZH material family. Furthermore, the absence of mirror symmetry across the monolayer plane allows the emergence of an out-of-plane piezoelectric response. The calculated *d*_31_ coefficient reaches 0.51 pm V^−1^ in SeWNH, which is larger than the values reported for MoSiGeN_4_ (−0.014 pm V^−1^),^13^ Ga_2_SSe (0.07 pm V^−1^),^39^ MoSiN_3_H (0.058 pm V^−1^).^42^ It is important to note that the calculated *d*_31_ coefficient characterizes the out-of-plane piezoelectric polarization induced strictly by applied in-plane stress. This should not be conflated with an out-of-plane mechanical deformation or out-of-plane stress response. The simultaneous presence of strong in-plane and out-of-plane piezoelectricity makes SeWNH monolayers attractive for a wide range of electromechanical applications in sensors, actuators, and energy-harvesting devices.

**Table 3:** Calculated bandgaps *E*_g_ using the PBE, HSE06, and PBE + SOC approaches, the valence-band spin splitting at the *K* point *λ*_v_, vacuum level difference Δ*Φ*, and work functions for the H/Se surfaces *Φ*_H_/*Φ*_Se_ of SeWZH monolayers. All values are in eV

	*E* ^PBE^_g_	*E* ^HSE06^_g_	*E* ^PBE+SOC^_g_	*λ*_v_	Δ*Φ*	*Φ*_H_	*Φ*_Se_
SeWNH	2.00	2.61	1.71	0.42	1.87	2.64	4.51
SeWPH	1.34	1.75	1.08	0.46	1.39	3.22	4.61
SeWAsH	1.20	1.59	0.92	0.49	1.50	3.09	4.59

### 3.3 Electronic properties

**Fig. 4:**
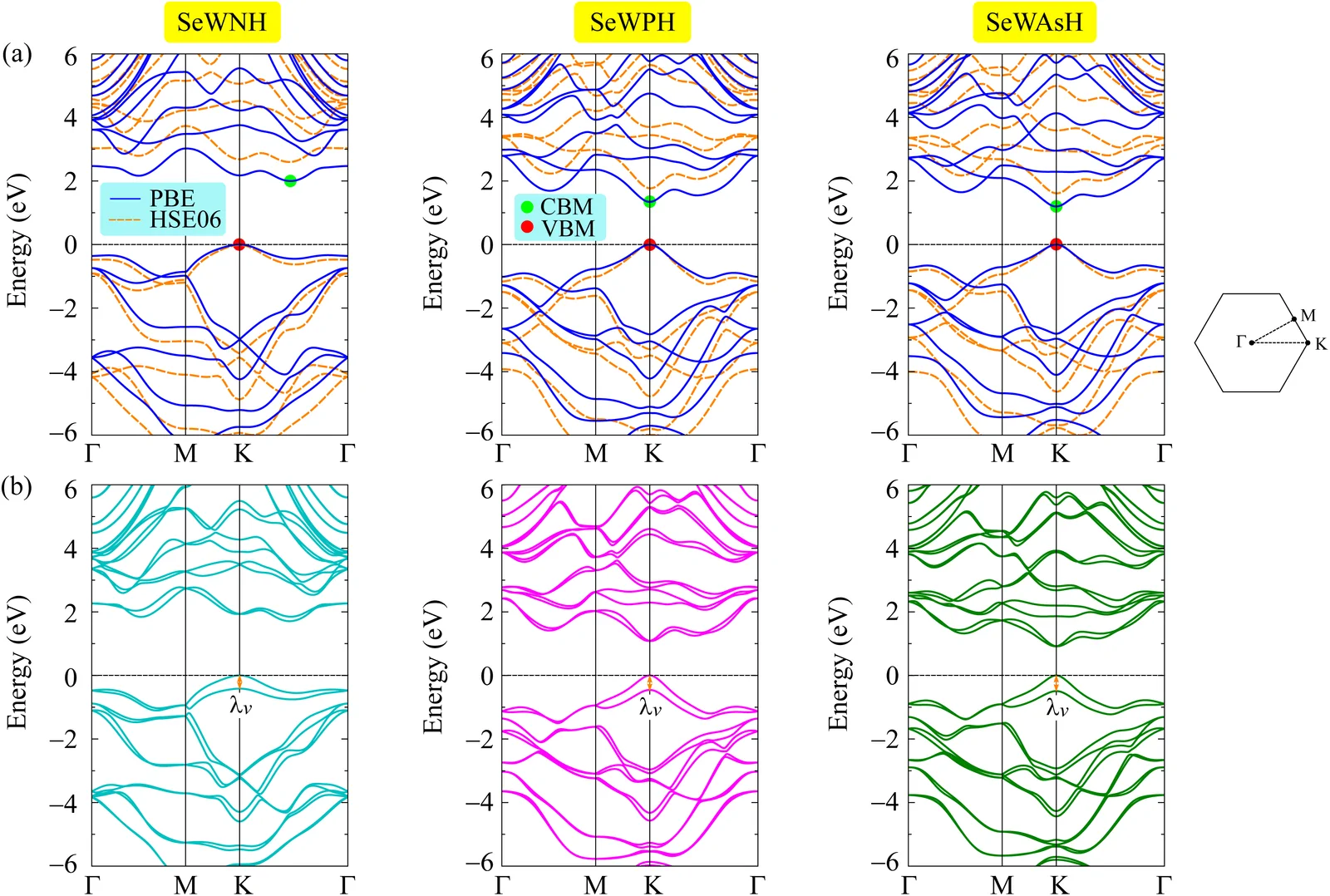
The electronic band structures of SeWNH, SeWPH, SeWAsH configurations from (a) PBE and HSE06, (b) PBE + SOC approaches. The first Brillouin zone is depicted on the right side of panel (a).

We next analyze the electronic band structures of the SeWZH monolayers to assess their potential for electronic and optoelectronic devices. The calculated PBE and HSE06 band dispersions along the *Γ* − *M* − *K* − *Γ* path are displayed in Fig. 4(a). All three compounds exhibit semiconducting electronic structures. At the PBE level, SeWNH shows an indirect transition of 2.00 eV with the valence-band edge located at *K* point and the conduction-band edge located between *K* and *Γ*. In comparison, SeWPH and SeWAsH are direct-semiconductors, where both band edges are located at the *K* point. Their band gaps are calculated to be 1.34 eV for SeWPH and 1.20 eV for SeWAsH as listed in Table 3. The gradual narrowing of the band gap from SeWNH to SeWAsH indicates enhanced electronic delocalization as the pnictogen atom becomes heavier. Hybrid-functional calculations using HSE06 reproduce the same electronic features and predict band gaps of 2.61, 1.75, and 1.59 eV for SeWNH, SeWPH, and SeWAsH, respectively. The calculated band-gap energies are suitable for visible-light absorption, indicating the potential of these monolayers for photovoltaic and optoelectronic applications.

The presence of heavy constituent atoms makes relativistic effects in the electronic behavior of SeWZH monolayers. Therefore, spin–orbit coupling is incorporated into the band-structure calculations. As illustrated in Fig. 4(b), SOC slightly modifies the electronic bands and results in a moderate narrowing of the band gaps to 1.71 eV for SeWNH, 1.08 eV for SeWPH, and 0.92 eV for SeWAsH as listed in Table 3. More importantly, SOC induces pronounced spin splitting around the *K* valley of the valence band due to the lifting of spin degeneracy. The calculated splitting energies *λ*_v_ are 0.42 eV, 0.46 eV, and 0.49 eV for SeWNH, SeWPH, and SeWAsH, respectively. The increasing trend of *λ*_v_ from N to As reflects the enhanced relativistic interaction associated with heavier pnictogen atoms.

**Fig. 5:**
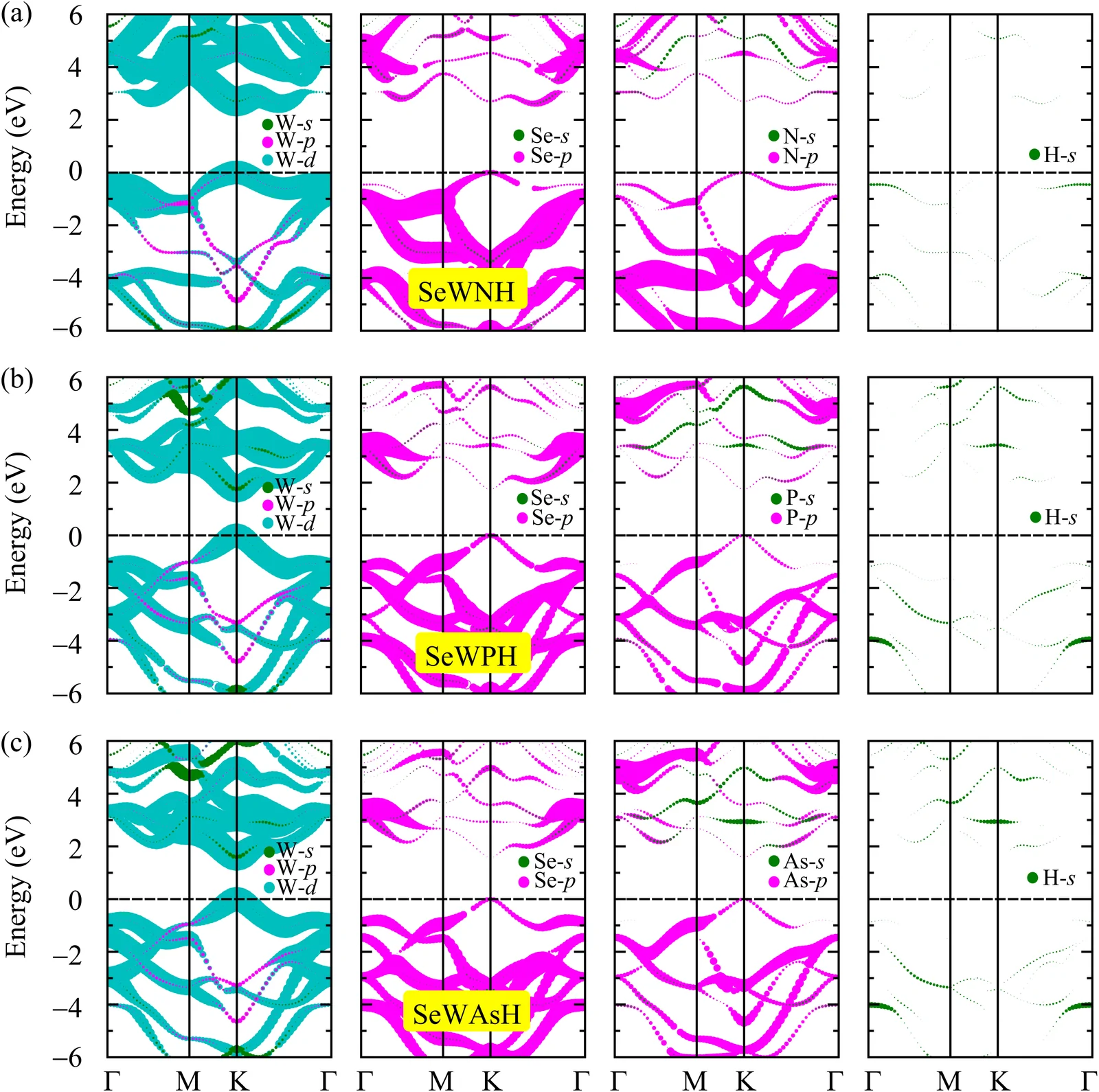
Weighted band structures of the SeWNH (a), SeWPH (b), and SeWAsH (c) materials calculated at the HSE06 level.

To further clarify the orbital origin of the electronic states, the weighted band structures of the SeWZH monolayers are analyzed and presented in Fig. 5. The results reveal that the conduction bands are dominantly composed of W-d states in all three systems. In addition, W-d orbitals contribute significantly to the valence-band region, particularly near the valence-band maximum of SeWZH. Meanwhile, the Se-p and Z-p orbitals are responsible for a substantial portion of the valence-band and conduction-band states. In comparison, the W-s, W-p, Se-s and Z-s states contribute only slightly to the band edges. The hydrogen contribution remains negligible because its electronic configuration contains only a single 1s state. Consequently, hydrogen mainly influences the electronic structure indirectly through bonding interactions with the neighboring pnictogen atoms, arising from the overlap between H-s and X orbitals.

**Fig. 6:**
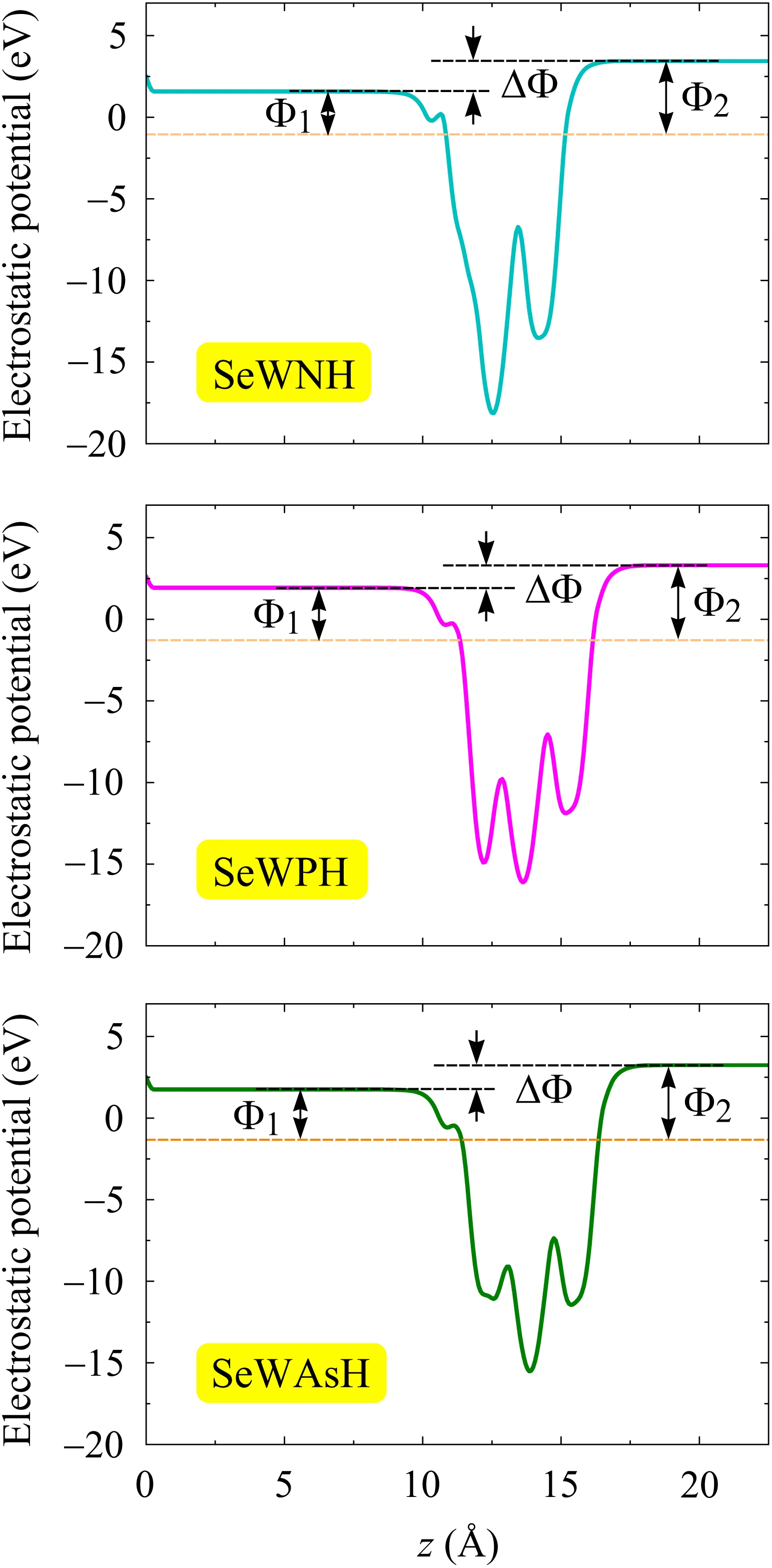
Planar average electrostatic potentials of the SeWZH (Z = N, P, As) monolayers calculated along the out-of-plane (*z*) direction. The differences in vacuum levels between the Se and H surfaces are indicated by Δ*Φ*.

The surface electronic characteristics of SeWZH monolayers are further examined through their work functions (*Φ*). The *Φ* value reflects the energy required for an electron to be emitted from the material into vacuum and can be obtained from the electrostatic potential profile as:

9 *Φ* = *E*_vac_ − *E*_F_.

where *E*_vac_ and *E*_F_ are respectively the energy at the vacuum and Fermi levels. Since, the two surfaces of Janus SeWZH are terminated by different atoms of Se and H, the charge distribution becomes intrinsically asymmetric. This asymmetry produces different vacuum levels on the Se and H sides, as shown in Fig. 6. To eliminate artificial electrostatic interactions caused by periodic boundary conditions, dipole corrections are applied during the calculations. According to Table 3, the calculated values on the Se side (*Φ*_Se_) fall within 4.51 to 4.61 eV, whereas the H-terminated surface exhibits considerably smaller *Φ*_H_ values of 2.64 to 3.22 eV. The larger *Φ*_Se_ indicates a higher electron extraction barrier from the Se surface compared with the H surface. The vacuum-level difference Δ*Φ* is determined from the work-function difference between these two sides. The Δ*Φ* value decreases from 1.87 eV for SeWNH to 1.39 eV for SeWPH and 1.50 eV for SeWAsH. The largest Δ*Φ* observed in SeWNH might originate from the strong electronegativity of nitrogen atom.

### 3.4 Transport properties

Finally, the charge-transport capability of the proposed Janus SeWZH monolayers is further characterized by evaluating their carrier mobility, which plays a key role in determining the efficiency of electronic transport and the overall performance of nanoelectronic and optoelectronic devices. To obtain transport coefficients, we utilize the AMSET package, which incorporates various important carrier–scattering mechanisms to enable mobility predictions.^19^ In particular, the effects of acoustic deformation potential (ADP), piezoelectric (PIE), ionized impurity (IMP), and polar optical phonon (POP) scattering are simultaneously considered. The overall mobility is then estimated according to Matthiessen's rule, which combines the contributions from individual scattering sources into the total mobility *µ*_total_:^22^10
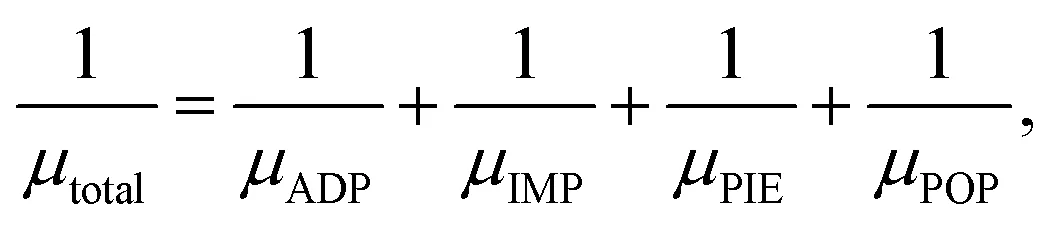
where *µ*_ADP_, *µ*_IMP_, *µ*_PIE_, and *µ*_POP_ correspond to the partial carrier mobility associated with the ADP, IMP, PIE, and POP scattering channels, respectively.

**Fig. 7:**
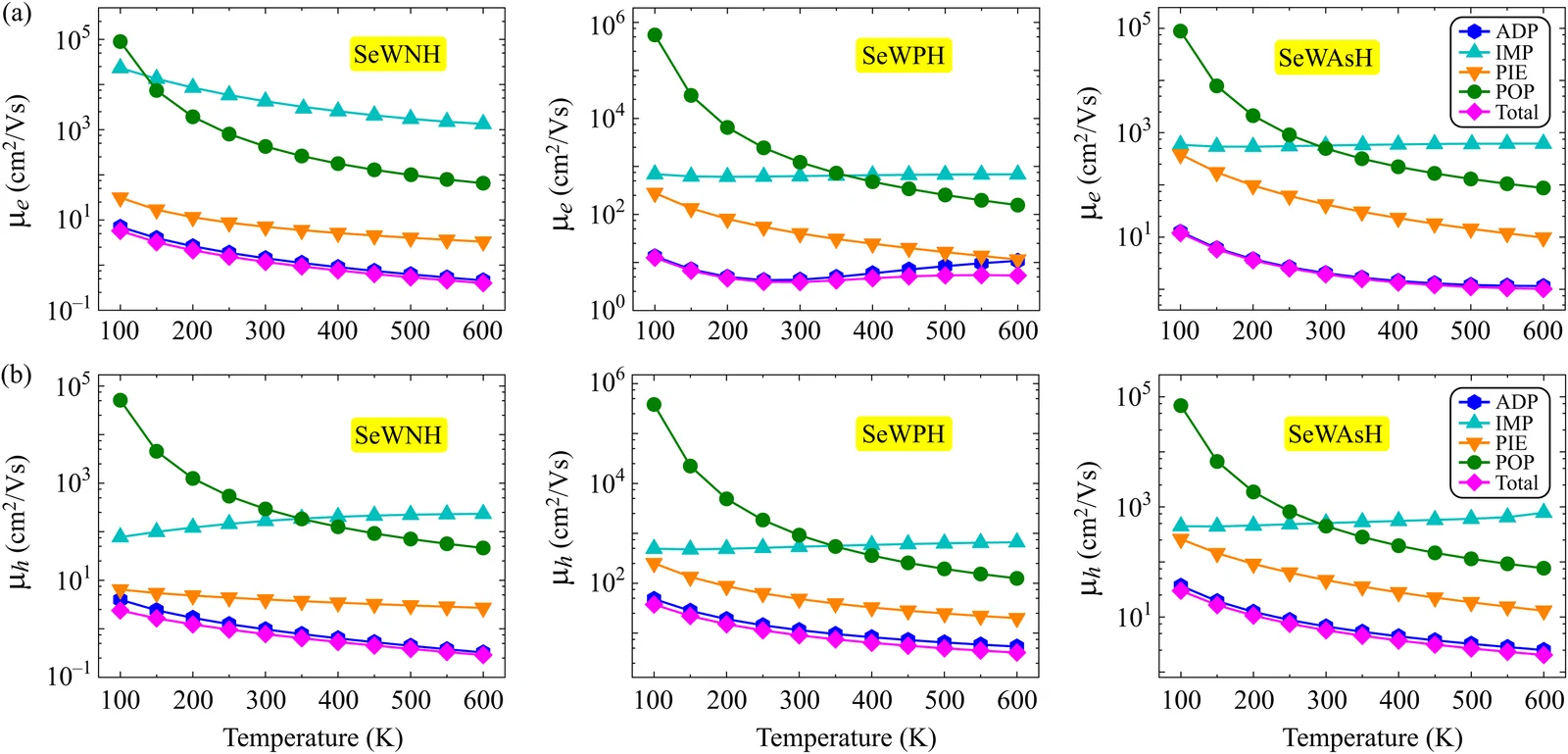
(a) Electron and (b) hole mobilities of SeWZH monolayers at low carrier concentration of 1 × 10^16^ cm^−3^.

The dependence of carrier mobility on carrier density has been previously investigated for MA_2_N_4_ systems, where mobility was found to remain relatively unaffected at low concentrations but decreased markedly once the carrier density reached the order of 10^19^ cm^−3^.^43^ Following this observation, we consider two representative carrier concentrations of 1 × 10^16^ cm^−3^ and 1 × 10^20^ cm^−3^, reflecting to low and high doped conditions. Carrier mobilities were calculated based on electronic structures obtained from the HSE06 hybrid functional. Fig. 7 presents the dependence of temperature on electron and hole mobilities of the SeWZH monolayers at a carrier concentration of 1 × 10^16^ cm^−3^. The analysis reveals that ADP scattering is the primary mechanisms governing carrier transport in SeWNH, SeWPH, and SeWAsH over the investigated temperature range of 100 to 600 K. The piezoelectric scattering also give secondary contribution to the carrier transport of the three monolayers. In contrast, the contribution from POP and IMP scattering remains relatively weak, particularly at lower temperatures. As temperature increases, the hole mobilities limited by ADP, PIE, and POP scattering gradually decrease, whereas the mobility associated with IMP scattering exhibits a different trend. Consequently, the overall carrier mobility decreases with increasing temperature. A similar trend is observed for the electron mobility, except in the case of SeWPH at temperatures between 300 and 600 K, where the total carrier mobility exhibits a slight increase due to the enhancement of the ADP-limited mobility. Among these investigated compounds, SeNPH has the lowest hole mobility of 0.78 cm^2^ V^−1^ s^−1^ at room temperature as tabulated in Table 4. Meanwhile, SeWPH possesses the highest hole and electron mobility of 9.05 and 3.90 cm^2^ V^−1^ s^−1^, while SeWAsH exhibits lower values of 5.74 and 1.93 cm^2^ V^−1^ s^−1^, respectively. These obtained values are comparable or higher than carrier mobility of 2D CrBNS_2_ (1.03 cm^2^ V^−1^ s^−1^)^44^ and T-ZrTe_2_ (4.7 cm^2^ V^−1^ s^−1^)^22^ materials, but still lower than other reported MoTe_2_ (39.2 cm^2^ V^−1^ s^−1^) and GeS (19.4 cm^2^ V^−1^ s^−1^).^45^

**Table 4:** Room-temperature carrier mobility *µ* in Janus SeWZH monolayers at different carrier concentrations *n*_c_. Total mobility is shown alongside contributions from individual scattering mechanisms

Carrier concentration (cm^−3^)	Carrier type	Compound	*µ*_ADP_ (cm^2^ V^−1^ s^−1^)	*µ*_IMP_ (cm^2^ V^−1^ s^−1^)	*µ*_PIE_ (cm^2^ V^−1^ s^−1^)	*µ*_POP_ (cm^2^ V^−1^ s^−1^)	*µ*_total_ (cm^2^ V^−1^ s^−1^)
1 × 10^16^	Electron	SeWNH	1.42	4.25 × 10^3^	7.08	424.00	1.18
		SeWPH	4.36	628.00	40.10	1220.00	3.90
		SeWAsH	2.04	568.00	42.10	498.00	1.93
	Hole	SeWNH	0.98	167.00	4.02	294.00	0.78
		SeWPH	11.50	539.00	48.50	921.00	9.05
		SeWAsH	6.73	508.00	46.70	447.00	5.74
1 × 10^20^	Electron	SeWNH	1.32	16.00	6.87	334.00	1.03
		SeWPH	13.20	36.80	19.20	343.00	6.33
		SeWAsH	1.63	23.60	11.90	174.00	1.34
	Hole	SeWNH	0.89	7.07	4.51	203.00	0.67
		SeWPH	10.20	26.00	35.00	264.00	5.92
		SeWAsH	4.82	21.10	21.00	146.00	3.23

**Fig. 8:**
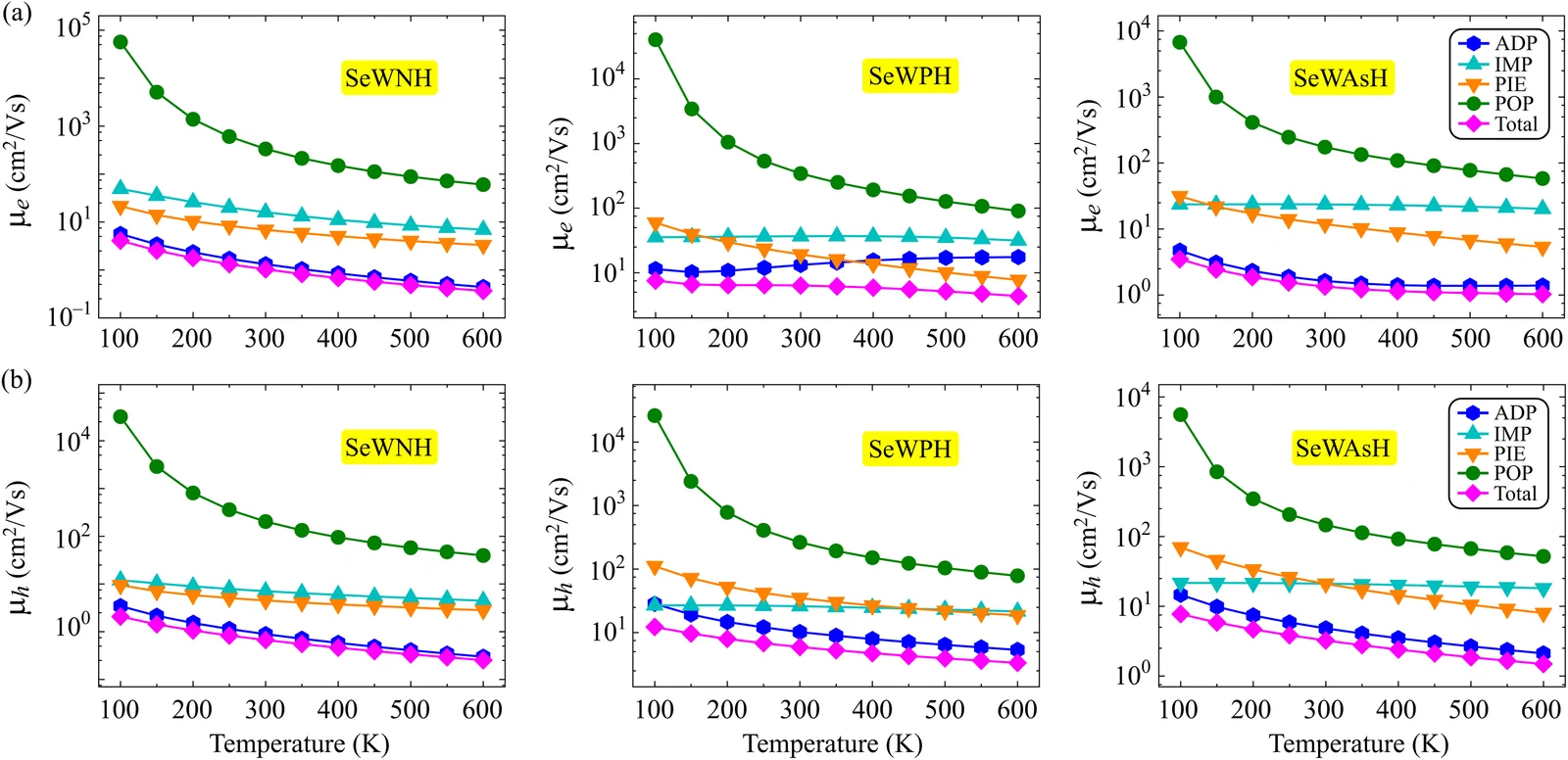
(a) Electron and (b) hole mobilities of SeWZH monolayers at high carrier concentration of 1 × 10^20^ cm^−3^.

The temperature-dependent carrier mobilities at the high carrier concentration of 1 × 10^20^ cm^−3^ are presented in Fig. 8. Similar to the low-concentration case, both hole and electron mobilities decrease progressively with increasing temperature. Among the scattering mechanisms considered, the role of POP scattering remains comparatively weak over the entire temperature range, particularly in the low-temperature region. In contrast, ADP scattering remains the primary factor limiting carrier mobility in all three SeWZH monolayers. The influence of PIE and IMP scattering to both electron and hole mobilities becomes increasingly important at the high carrier concentration. At 300 K, the electron mobilities of SeWNH, SeWPH, and SeWAsH are predicted to be 1.03, 6.33, and 1.34 cm^2^ V^−1^ s^−1^, respectively. The corresponding hole mobilities are 0.67 cm^2^ V^−1^ s^−1^ for SeWNH, 5.92 cm^2^ V^−1^ s^−1^ for SeWPH, and 3.23 cm^2^ V^−1^ s^−1^ for SeWAsH. The calculated results reveal that carrier transport in Janus SeWZH monolayers is intrinsically limited, leading to relatively low carrier mobilities. Such behavior is consistent with numerous recently proposed two-dimensional semiconductors such as T-PtS_2_,^22^ h-BP,^46^ and WBNSe_2_.^47^

## 4 Conclusion

Overall, this study presents a detailed first-principles prediction of the Janus SeWNH, SeWPH, and SeWAsH monolayers, focusing on their stability, electronic structure, piezoelectricity and charge-transport behavior. The calculated results verify that all proposed structures are dynamically and structurally stable, supporting their potential synthesis in future experiments. The SeWNH systems exhibits semiconducting characteristics with indirect bandgap of 2.61 eV at HSE06 level. The SeWPH and SeWAsH monolayers are predicted to possess direct HSE06 band gaps of 1.75 and 1.59 eV, respectively. Such band-gap energies are well suited for visible-light harvesting, indicating their potential usefulness in photovoltaic and optoelectronic devices. In addition, the spin–orbit coupling introduces slightly modifications to electronic states of the three monolayers, SOC induces spin splitting around the *K* valley of the valence band due to the lifting of spin degeneracy. Notably, the intrinsic lack of mirror symmetry allows the SeWZH monolayers to exhibit piezoelectric effects in both the in-plane and out-of-plane directions. Particularly, the predicted out-of-plane piezoelectric coefficient of SeWNH is competitive with those reported for other 2D materials. The simultaneous presence of strong in-plane and out-of-plane piezoelectricity makes SeWNH monolayers attractive for a wide range of electromechanical applications in sensors, actuators, and energy-harvesting devices. Further examination on the carrier transport process shows that acoustic deformation potential scattering is the major contributor to mobility degradation. The strong influence of this scattering mechanism leads to comparatively low carrier mobilities, indicates the important role of intrinsic carrier–phonon interactions in determining the transport performance of these materials. Consequently, this work provides valuable insights into the physical properties of Janus SeWZH monolayers and demonstrates their potential for multifunctional applications in nanoelectronics, spintronics, and energy-harvesting technologies.

## Conflicts of interest

The authors declare that they have no conflicts of interest.

## Data Availability

The data that support the findings of this study are available from the authors upon reasonable request.
